# Supplementary figures and images for: What the hippocampus tells the HPA axis: Hippocampal output attenuates acute stress responses via disynaptic inhibition of CRF+ PVN neurons
Source: Neurobiol Stress. 2022 Aug 5;20:100473. doi: 10.1016/j.ynstr.2022.100473 (PMC9379952; doi:10.1016/j.ynstr.2022.100473)

# Supplemental Figure 1

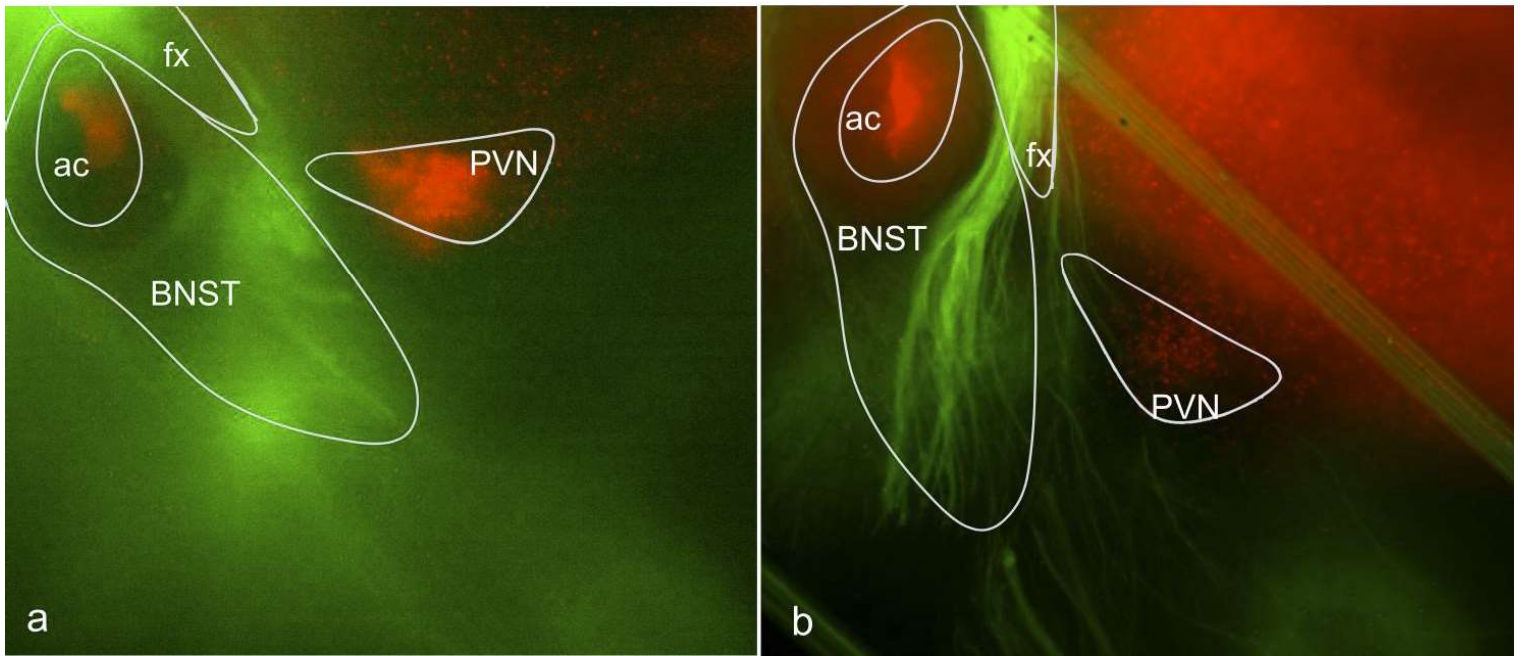

Supplement: Multimedia component 1 [file mmc1.pdf]
